# The Influence and Compensation of Process on Measurement Accuracy in Digital Grating Focusing and Leveling Sensors

**DOI:** 10.3390/mi16121326

**Published:** 2025-11-26

**Authors:** Shiguang Li, Xianjie Li, Guocai He

**Affiliations:** 1 Institute of Microelectronics of the Chinese Academy of Sciences, Beijing 100029, China; 2 Jiangsu Yingsu Integrated Circuit Equipment Co., Ltd., Xuzhou 221300, China; jacklee@ysphotech.com (X.L.); hegc@ysphotech.com (G.H.)

**Keywords:** digital grating, focusing and leveling, process pattern, Criminisi algorithm, image restoration

## Abstract

The digital grating focusing and leveling sensor is a kind of wafer height sensor for focus control in a lithography tool by measuring the displacement of an optical grating image reflected from the wafer surface. The process pattern on the wafer surface can significantly affect the measurement accuracy of the sensor. To mitigate this effect, the Criminisi algorithm for image processing is employed. First, process patterns in the optical grating image are identified and masked with a specific color—yellow in this paper. The Criminisi algorithm is then applied to recover the clear image in the masked region. To evaluate the algorithm performance, 50 masked images are recovered and compared with the original clear image where the mask ratios range from 1% to 15%. The experimental results indicate that the mean repair accuracy is below 1 nm after 10 repair iterations for a given mask ratio and the maximum error in a single repair is 68 nm across all 50 images.

## 1. Introduction

As the technology nodes of integrated circuits continue to shrink, focus control in a lithography tool has become crucial. High-precision focus control is necessary for ensuring pattern quality and final chip yield. When performing focus control, a key hardware is a focusing and leveling sensor. The purpose of the sensor is to provide real-time feedback for focus control by measuring the defocus value of the wafer surface relative to the optimal focal plane of the projection optics. The measurement accuracy of the focusing and leveling sensor determines the focus control accuracy of the lithography tool. To achieve high-precision measurement, lithography manufacturers like ASML and Nikon employ highly complex optical systems [1,2]. Scholars from the Institute of Microelectronics of the Chinese Academy of Sciences have developed a focusing and leveling sensor based on digital gratings [3,4]. The device features a simple structure and is capable of measuring the height of specular reflective objects, such as bare wafers. Under controlled environmental conditions, it achieves a measurement resolution of 10 nm and an accuracy of the order of tens of nanometers [5]. However, the presence of IC process patterns, such as photoresist or other micro-structures, degrades the measurement accuracy. This influence is called process dependency [2,6]. Any optical sensor has process dependency to some extent. The root cause is that the beam propagation is disturbed by the wafer’s surface characteristics. This paper aims to investigate a compensation method to address the sensor’s process dependency.

## 2. The Influence Mechanism of Process Pattern on Measurement Accuracy

**Figure 1:**
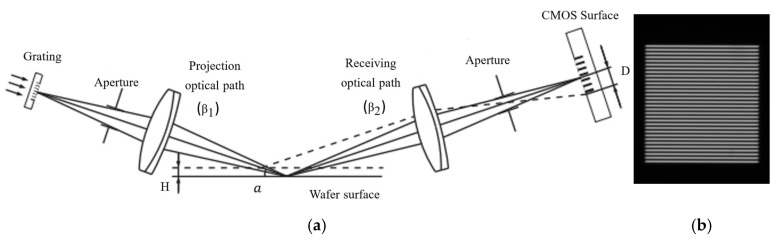
Digital grating focusing and leveling sensor. (**a**) Schematic diagram; (**b**) Grating image on the camera.

The schematic diagram of the focusing and leveling sensor based on a digital grating is shown in Figure 1a, which is mainly composed of a light source, an optical grating, a projection optical path, a receiving optical path, and a digital camera. The grating is imaged onto the wafer surface, and after being reflected by the wafer, it is imaged again onto the camera, as shown in Figure 1b.

The wafer surface height variation H results in the displacement D of the grating image on the camera, and the relationship between H and D is as follows according to the triangular principle:

(1) $$H=\frac{D}{2\beta_{2}\cos\alpha }$$

where α is the angle between the incident light and the wafer surface, and $\beta_{1}$, $\beta_{2}$ is the magnification of the projection path and the receiving path respectively. α is 22° and $\beta_{2}$ is 1.16× in this paper.

**Figure 2:**
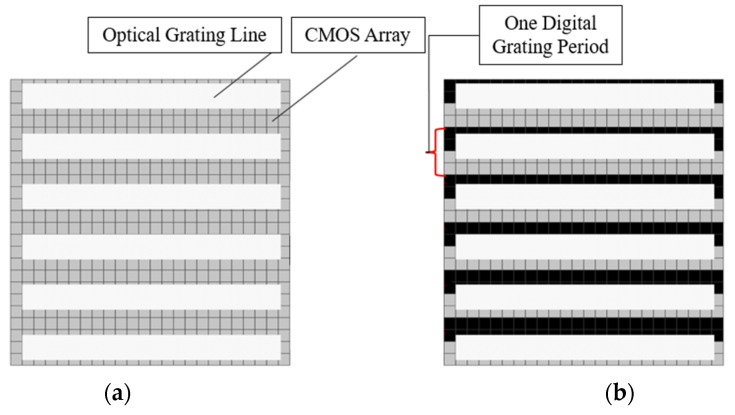

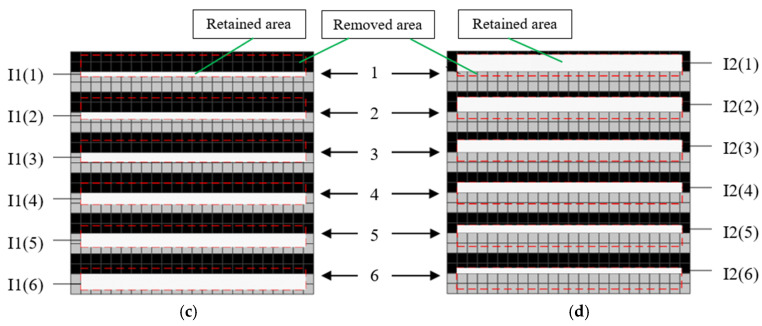
(**a**) Optical grating image; (**b**) Digital grating construction; (**c**) Digital grating 1; (**d**) Digital grating 2. The red dashed rectangle represents the optical grating line.

**Figure 3:**
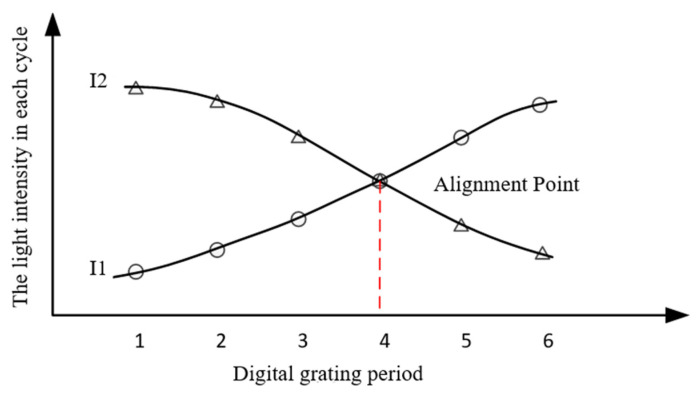
Integrated light intensity curves I1 and I2. The red dashed line indicates the position of the alignment point in the digital grating periods.

In order to obtain the H value with nanometer accuracy, the D value must be as accurate as possible according to Equation (1). This sensor applies a patented digital image processing technology to obtain the high-precision D [7]. The principle is as follows [8]: Figure 1b is schematically represented as Figure 2a, where the white rectangles represent the bright lines of the grating and the small gray squares represent the pixels of the photosensitive chip (CCD or CMOS). By periodically retaining or removing the light intensity signal on each pixel of Figure 2a, the photosensitive chip surface can be constructed as two complementary digital gratings as shown in Figure 2b. A pixel group consisting of two rows of black areas and two rows of gray areas represents a digital grating period. When the black area represents that the light intensity signal on the pixels is removed and the gray area represents that the light intensity signal is retained, a first digital grating is formed in Figure 2c. When the black area represents that the light intensity signal is retained, and the gray area represents that the light intensity signal is removed, another digital grating is formed in Figure 2d. In this paper, these two gratings are called digital gratings 1 and 2, respectively. The period of the digital grating is T. T can only be an even number of the pixel. In Figure 2b, T = 4. After the above periodic retention or removal of the light intensity signal, a complete optical grating image is divided into two grating images with complementary light intensities. Assuming that the period of the optical grating image is T + ∆T, a grating difference structure is formed with the optical grating image and the digital grating, similar to a vernier caliper. ∆T is the direct measurement resolution. When ∆T is less than the size of a pixel, the image resolution is subdivided. In practice, in order to precisely measure the alignment point of this “optical vernier caliper”, the retained light intensity in each digital grating period is integrated. For Figure 2c, the light intensity integral value of each period (gray area) is I1(1)–I1(6), and for Figure 2d, the light intensity integral value of each period (black area) is I2(1)–I2(6). All values of I1 and I2 are plotted as a curve, as shown in Figure 3. By fitting I1 and I2, we can obtain the intersection point, which represents the point where the light intensity of I1 and I2 is equal, and which is defined as the alignment point. When the grating is displaced, the position of the alignment point changes. According to the vernier caliper principle, the optical grating image moves a period difference ∆T, and the alignment point moves a digital grating period T; therefore, the grating displacement D is then as follows:

(2) $$D=\frac{T_{x}\times ∆T}{T}$$

where $T_{x}$ is the period number of the digital grating for the alignment point movement. For Figure 1b, the period of the optical grating image is 9.6417 pixels, the digital grating period T is 10 pixels, and the period difference ∆T between them is 0.3583 pixels.

**Figure 4:**
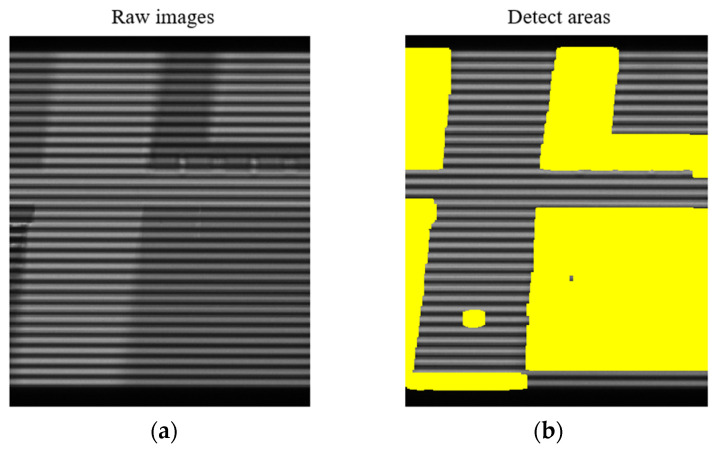
(**a**) Raw image affected by the pattern on the wafer; (**b**) The identified region to be repaired as shown in yellow.

It can be seen from the calculation process that the accuracy of the alignment point position is greatly dependent on the light intensity curves I1 and I2. For an ideal specular reflective object, the optical grating pattern is an ideal periodic pattern, as shown in Figure 1b. The alignment point position calculated based on the I curve can reach nanometer level. However, for the grating images affected by process patterns, as shown in Figure 4a, the I curve is seriously deformed, making it difficult to find the accurate alignment point position. In severe cases, the shape of the I curve cannot be clearly seen. Therefore, the image region affected by process must be eliminated. Reference [9] segments the image to target such defects or patterns as stains, particles, scratches, and grooves on the wafer surface, and replaces the light intensity value in the abnormal region with that in the normal region, achieving a good compensation effect. This paper investigates another image restoration algorithm to compensate for the process pattern effects.

## 3. Compensation Algorithm Design and Implementation

To compensate for the process dependency, the first step is to identify the image regions disturbed by the process patterns. By comparing Figure 1b and Figure 4a, and subtracting the two images, the region requiring repair can be identified using a threshold method, as shown by the yellow regions in Figure 4b. The following section describes the algorithm used to restore the image in these yellow areas.

**Figure 5:**
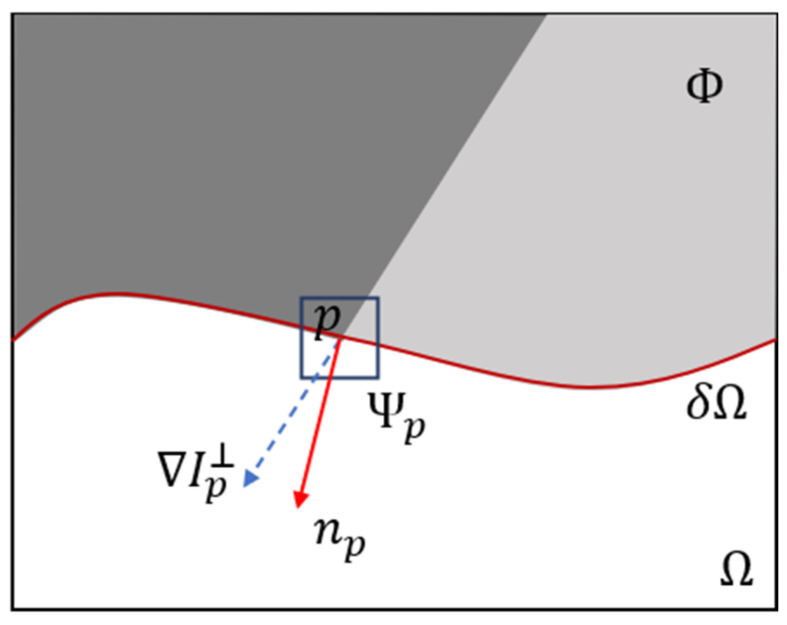
Schematic diagram of Criminisi algorithm.

Traditional image restoration techniques, such as inverse filtering, Wiener filtering, Luck–Richardson restoration, and blind deconvolution, primarily focus on restoring blurred images to a clearer state [10,11]. These methods, however, have significant limitations in addressing the problem presented in this paper. Criminisi et al. first proposed an exemplar-based inpainting algorithm [12]. Figure 5 is a schematic diagram of the principle of the Criminisi algorithm [12]. The Ω below the red curve represents the target region to be repaired, the Φ above the red curve represents the source region which need not repair, and the red curve δΩ represents the boundary between them. The dark gray and light gray colors inside the Φ represent two intensity values. p is a point on δΩ. The area inside the blue square $\Psi_{p}$ represents the sample block centered at p which is to be repaired. The red arrow $n_{p}$ is the unit normal vector of δΩ at p, and the blue arrow $\nabla I_{p}^{⊥}$ is the isophote (direction and intensity). The $n_{p}$ and $\nabla I_{p}^{⊥}$ are both directed to the target region Ω.

The algorithm implementation steps are as follows:

(1) Priority strategy

The priority formula of the Criminisi algorithm is as follows:

(3) $$P\left(p\right)\;=\;C\left(p\right)\times D\left(p\right)$$

where C(p) represents the confidence term of the sample block at point p. D(p) is the data term which is a function of the strength of isophotes hitting the front δΩ at each iteration. The calculation formula of C(p) and D(p) is as follows [12]:

(4) $$C\left(p\right)\;=\;\frac{\sum_{q\in \left(\Psi_{p}\cap \Phi \right)}\tau \left(q\right)}{\left|\Psi_{p}\right|}$$

(5) $$D\left(p\right)=\frac{\left|\nabla I_{P}^{⊥}\cdot n_{p}\right|}{L}$$

**Figure 6:**
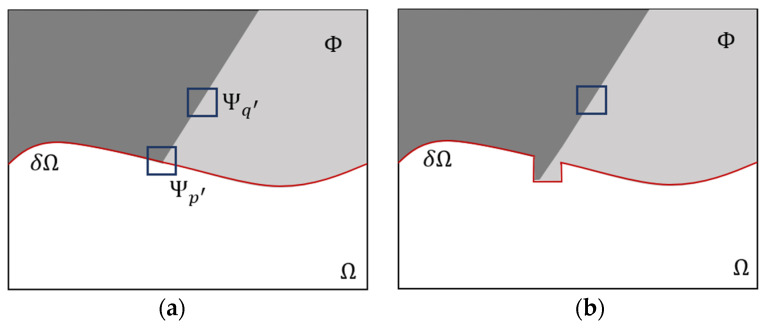
Repair process of a sample block: (**a**) find the priority point p’ and the best-matching block ${\;\Psi }_{q^{\prime }}$, and (**b**) copy ${\;\Psi }_{q^{\prime }}$ to $\Psi_{p^{\prime }}$.

Here, $\left|\Psi_{p}\right|$ represents the number of pixels in the sample block and q is the intersection block of the sample block $\Psi_{p}$ and the source region Φ. τ = 1 for the pixels in the q area. The physical meaning of C(p) is the ratio of the q area to the area of the sample block. The larger of C(p), the greater the proportion of known information contained in $\Psi_{p}$, suggesting it should be repaired first. L is the normalization factor, which is usually 255 for a gray image [13]. By calculating P(p) at all points along δΩ, the p with maximum P is found, and is denoted as p′ in Figure 6a [12].

(2) Searching and filling the best-matching block

After the p′ is found, the self-similarity algorithm of the image is used to find the best-matching block in source region Φ to fill in the sample block $\Psi_{p^{\prime }}$. If q represents any point in Φ, ${\;\Psi }_{q}$ represents the matching block centered on q. The size of ${\;\Psi }_{q}$ and $\Psi_{p^{\prime }}$ is same. The best-matching block $\Psi_{q^{\prime }}$ is found by minimizing the Sum of Squared Difference (SSD) between $\Psi_{p^{\prime }}$ and ${\;\Psi }_{q^{\prime }}$. Then $\Psi_{q^{\prime }}$ is copied to $\Psi_{p^{\prime }}$, as shown in Figure 6b. The search algorithm is as follows [12]:

(6) $$\Psi_{q^{\prime }}=\arg\underset{\Psi_{q}\in \Phi }{\min}\mathrm{SSD}\left(\Psi_{p^{\prime }}\;,{\;\Psi }_{q}\right)$$

The SSD model for finding the best-matching block is as follows [14]:

(7) $$\mathrm{SSD}\left(\Psi_{p^{\prime }},\Psi_{q}\right)=\sum \left[{\left({p^{\prime }}_{\left(i,j\right)}^{R}-q_{\left(i,j\right)}^{R}\right)}^{2}+{\left({p^{\prime }}_{\left(i,j\right)}^{G}-q_{\left(i,j\right)}^{G}\right)}^{2}+{\left({p^{\prime }}_{\left(i,j\right)}^{B}-q_{\left(i,j\right)}^{R}\right)}^{2}\right]$$

where p^R^, p^G^, p^B^ and q^R^, q^G^, q^B^ are the three primary color (RGB) intensities of the sample block and the matching block, respectively. i and j are the pixel numbers in the row and column respectively in $\Psi_{p^{\prime }}$ and ${\;\Psi }_{q}$.

(3) Dynamic update of confidence term

After replacing $\Psi_{p^{\prime }}$ with $\Psi_{q^{\prime }}$, the boundary δΩ will change. The new boundary after matching-block filling is shown as red line in Figure 6b. The confidence term needs to be updated. The update equation is shown below.

(8) $$C\left(p\right)=C\left(p^{\prime }\right),\forall p\in \Psi_{p^{\prime }}\cap \Omega$$

Repeat the above steps until all the target region Ω is repaired. 

**Figure 7:**
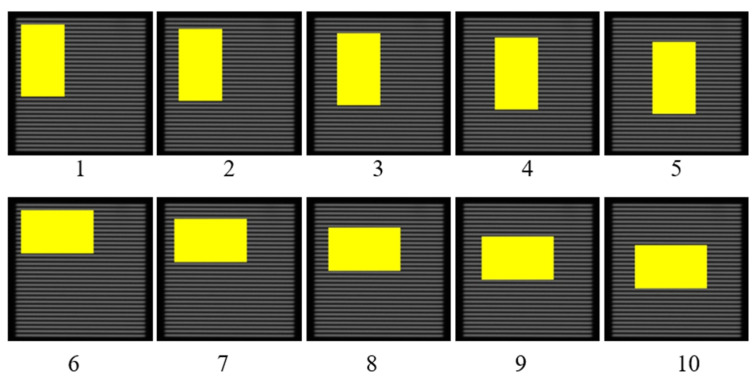
Images to be repaired with mask area ratio of 15%. There is no pattern inside the yellow masks. The mask location of other ratios is the same.

**Figure 8:**
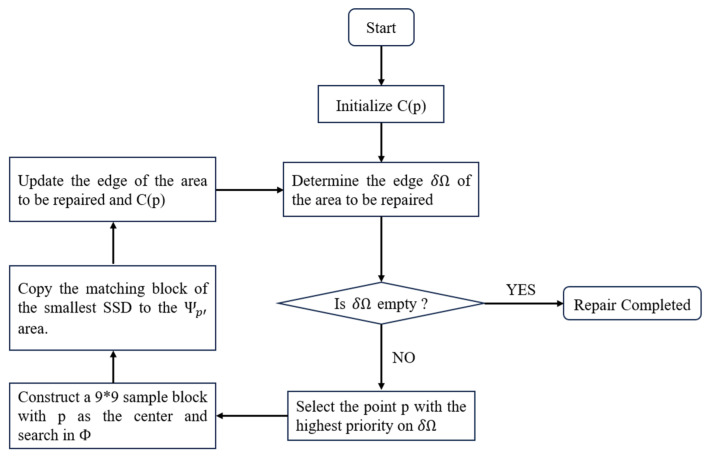
Flow chart of the Criminisi algorithm.

This paper preliminarily simulates the processed pattern on the wafer by covering the grating image in Figure 1b with a rectangular mask, which represents Ω. Such simulation helps to first investigate the effectiveness of the algorithm. Future work will process the real patterns like that in Figure 4. Experiments are conducted with five mask area ratios (1%, 3%, 5%, 10%, 15%) to evaluate the repair effect. For each mask area ratio, the rectangle is placed vertically and horizontally respectively at five positions in Figure 7. The total repair experiments are thus conducted 50 times. The experiment applies MATLAB 2018b to implement the algorithm, and the repair flow is shown in Figure 8 [13].

## 4. Repair Effect and Performance Analysis

**Figure 9:**
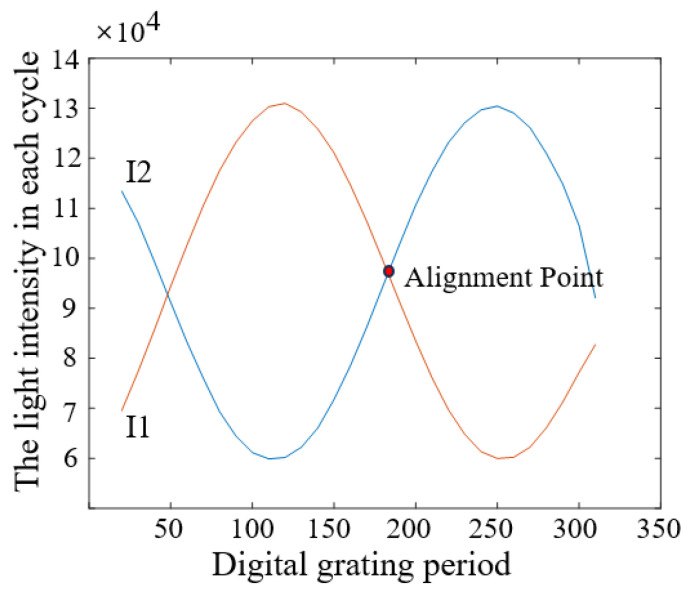
Integrated light intensity curve in Figure 1b.

**Figure 10:**
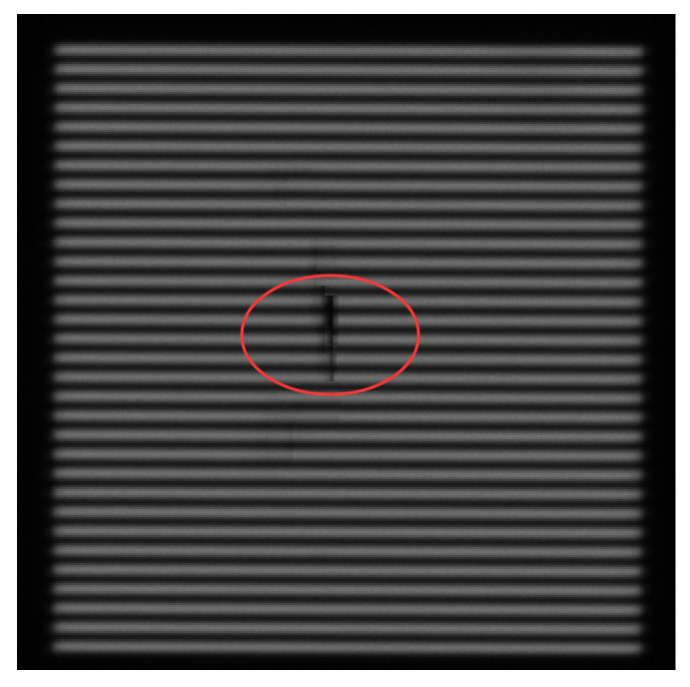
The image after repair for the 5th image in Figure 7. A slight repair defect appears as circled with red line.

The repair accuracy of the image restoration algorithm is evaluated with the image in Figure 1b and Equations (1) and (2). The evaluation procedure is conducted as follows: At first, an intensity curve similar to that in Figure 3 is generated in Figure 9 for the original image, and the alignment point position is 184.1504 pixel. This value is listed as the reference (“Ref”) in Table 1. There are two alignment points in Figure 9 and the right one is selected. The 50 masked images like those in Figure 7 are then processed using the repair algorithm to reconstruct the clear optical grating image, i.e., the image in the yellow region is recovered. After restoration, the alignment point positions are calculated for each repaired image. For example, the alignment positions for the repaired images in Figure 7 are 184.1594–184.0578 pixels, respectively, as listed in the rightmost column in Table 1. Since the repaired images originated from the same image, the alignment point position should ideally remain unchanged after restoration. Any deviation in the alignment point position is therefore attributed to the restoration algorithm. This deviation, denoted as T_x_ in Equation (2), represents the repair error in pixel units. T_x_ is converted into the displacement error D of the grating image, and subsequently into the height error H in the Z direction via Equation (1). The results indicate that the mean repair accuracy of the 10 images for each mask ratio was below 1 nm in the Z direction, while the maximum repair error observed among all 50 samples was 68 nm, which is for the 5th image in Figure 7. There is a slight defect after repair as shown in Figure 10. This can be repaired second time to meet the requirements. From Table 1, it can be seen that the repair uncertainty is more serious when the mask ratio is greater.

## 5. Conclusions

This paper investigates the influence of process patterns on the measurement accuracy of a nanometer degree digital grating focusing and leveling sensor. To mitigate this effect, a compensation scheme based on the Criminisi algorithm is proposed for repairing the degraded optical grating images in this sensor. The average repair accuracy of 10 images at different positions for a certain mask ratio was below 1 nm in the Z direction, and the maximum repair error is 68 nm across 50 test images. Future work will focus on enhancing the algorithm’s real-time performance and its adaptability to complex patterns.

## Figures and Tables

**Table 1:** Experimental data to evaluate the repair accuracy (error) of Criminis algorithm.

	Mask Area	Ref	1%	3%	5%	10%	15%
Items							
Position 1 in Figure 7		184.1504	184.1525	184.1502	184.1524	184.1422	184.1594
2			184.1502	184.1542	184.1515	184.1467	184.1912
3			184.1511	184.1260	184.1201	184.1073	184.0910
4			184.1499	184.1589	184.1766	184.0786	184.1491
5			184.1513	184.1405	184.0990	184.2744	184.9940
6			184.1504	184.1510	184.1447	184.1581	184.1725
7			184.1486	184.1511	184.1635	184.1403	184.1630
8			184.1501	184.1511	184.1595	184.1738	184.0880
9			184.1516	184.1539	184.1425	184.1716	183.3362
10			184.1537	184.1503	184.1497	184.0412	184.0578
Mean alignment point (pix)			184.1509	184.1487	184.1460	184.1434	184.1402
Error in alignment Tx (pix)			0.0005	−0.0038	−0.0043	−0.0090	−0.0020
Error of D (nm)			0.0860	−0.6535	−0.7395	−1.5479	−0.3440
Mean error in Z direction H (nm)			0.0400	−0.3038	−0.3438	−0.7196	−0.1599
Max (pix)			184.1537	184.1589	184.1766	184.2744	184.9940
Min (pix)			184.1486	184.1260	184.0990	184.0412	183.3362
Error in alignment Tx (pix)		Ref-Max	−0.0033	−0.0064	−0.0264	−0.1220	−0.8518
		Ref-Min	0.0018	0.0265	0.0512	0.1112	0.8060
Error of D (nm)		Ref-Max	−0.5675	−1.1007	−4.5404	−20.9820	−146.4960
		Ref-Min	0.3096	4.5576	8.8056	19.1246	138.6191
Error in Z direction H (nm)		Ref-Max	−0.2638	−0.5117	−2.1108	−9.7543	−68.1039
		Ref-Min	0.1439	2.1188	4.0936	8.8908	64.4420

## Data Availability

The original contributions presented in this study are included in the article. Further inquiries can be directed to the corresponding author.
